# Examining changes in sexual lifestyles in Britain between 1990–2010: a latent class analysis approach

**DOI:** 10.1186/s12889-024-17850-1

**Published:** 2024-02-03

**Authors:** Luke Muschialli, Pantelis Samartsidis, Anne M. Presanis, Catherine H. Mercer

**Affiliations:** 1 UCL Medical School, Faculty of Medical Sciences, University College London, London, UK; 2 Medical Research Council Biostatistics Unit, School of Clinical Medicine, University of Cambridge, Cambridge, UK; 3 Centre for Population Research in Sexual Health and HIV, Institute for Global Health, University College London, London, UK

**Keywords:** Epidemiology, Sexual and reproductive health, Mixture modelling, Behaviour

## Abstract

**Background:**

Understanding sexual lifestyles and how they change over time is important for determining the likelihood of sexual health outcomes. Standard descriptive and regression methods are limited in their ability to capture multidimensional concepts such as sexual lifestyles. Latent Class Analysis (LCA) is a mixture modelling method that generates a categorical latent variable to derive homogenous groups from a heterogeneous population. Our study investigates (1) the potential of LCA to assess change over time in sexual lifestyles and (2) how quantifying this change using LCA compares to previous findings using standard approaches.

**Methods:**

Probability-sampled data from three rounds of the National Survey of Sexual Attitudes and Lifestyle (Natsal) were used, restricted to sexually active participants (i.e., those reporting sexual partners in the past year) aged 16–44 years (N_1990_ = 11,738; N_2000_ = 9,690; N_2010_ = 8,397). An LCA model was built from four variables: number of sexual partners (past year), number of partners without a condom (past year), age at first sex and self-perceived HIV risk. Covariates included age, ethnicity, educational attainment, same-sex attraction, and marital status. Multinomial regression analyses and Chi-Squared tests were used to investigate change over time in the size of each class.

**Results:**

We successfully used a LCA approach to examine change in sexual lifestyle over time. We observed a statistically significant increase between 1990 and 2010 in the proportion of men (χ^2^ = 739.49, *p* < 0.01) and women (χ^2^ = 1270.43, *p* < 0.01) in a latent class associated with reporting 2 or more partners in the last year, relatively high probabilities of reporting condomless sex partners, greater self-perceived HIV risk, and a high probability of first sex before age 16 years, increasing from 19.5% to 31.1% (men) and 9.9% to 22.1% (women).

**Conclusion:**

Our results indicate the viability of LCA models to assess change over time for complex behavioural phenomena. They align with previous findings, namely changing sexual lifestyles in Britain in recent decades, partnership number driving class assignment, and significant sex differences in sexual lifestyles. This approach can be used to extend previous LCA models (e.g., to investigate the impact of COVID-19 on sexual lifestyles) and to support empirical evidence of change over time, facilitating more nuanced public health policy.

**Supplementary Information:**

The online version contains supplementary material available at 10.1186/s12889-024-17850-1.

## Background

Sexual and Reproductive Health (SRH) is a public health priority [1] and sexual lifestyles, that is, the context and drivers of sexual attitudes and behaviours, are major determinants of SRH [2]. There is heterogeneity in sexual lifestyles within the general population [3–5], including by sex and age-group [2, 6, 7]. In recent decades, significant changes in sexual lifestyles have been observed in Britain, as well as in other high-income countries, reflecting in part a trend in liberalising attitudes towards sex and sexuality [7]. Changing sexual lifestyles have important implications for public health, as determining trajectories of change is an effective strategy for identifying groups most in need of intervention [8] and ensuring that interventions are targeted using the most clinically efficacious approach to result in behaviour change [9]. Sexual behaviours, relationship factors and responses to measures that prevent sexually transmitted infections (STIs) and unintended pregnancy (e.g., use of condoms) intersect, creating complex individual sexual lifestyle ‘profiles’, meaning that investigating individual factors and their possible complex interactions in a standard descriptive or regression analysis [10] may not adequately identify those most vulnerable to adverse SRH outcomes [11, 12].

Latent Class Analysis (LCA) is a mixture-modelling technique used to classify individuals into a discrete set of groups, using an unobserved categorical variable, where individuals within classes share similar characteristics. The latent variable is estimated based on multivariate clustering of measured (manifest) variables, accounting for population heterogeneity. Each individual is assigned to a class, based on their highest estimated probability of class membership [13, 14], with an underlying assumption that covariation between manifest variables is explained by the latent variable [13, 14]. The number of these classes and their sizes are not known a priori, but can be chosen using model-fit statistics [15]. LCA has several advantages over similar cluster analysis techniques (e.g., k-means), including greater use of formal criteria to decide upon a final model and flexibility in accommodating different variable scales [4], which is useful when analysing survey data [15]. LCA also addresses some of the methodological challenges of traditional subgroup analysis, such as high type I error, low statistical power and a difficulty analysing multidimensional interactions [13]. Although similar results could be obtained through modelling techniques such as logistic regression, this can be complicated by collinearity and the need to model complex, multi-dimensional interactions between manifest variables [5]. LCA can be used to highlight nuanced patterns of sexual lifestyle [16] forming a more complete picture of an individual’s sexual profile [4, 17] which can help streamline the design and delivery of interventions [13]. LCA has previously been used in the context of SRH [3–5, 16], but to our knowledge, a LCA approach has not been used to investigate changes over time in sexual lifestyles. This is important for understanding current, and informing future, SRH needs and the changing dynamics of the population as a whole, as well as vulnerable communities within the population.

Our paper therefore has two aims: firstly to investigate the use of LCA to capture change over time in sexual lifestyles, and secondly, to then ascertain both the extent of change over time and the sociodemographic correlates of these changes, comparing the findings using LCA to those from using standard regression techniques and empirical evidence.

## Methods

### Data

The National Survey of Sexual Attitudes and Lifestyles (Natsal) is a nationally representative survey of sexual behaviour, its context, drivers and consequences, undertaken approximately decennially with the British general population. The survey has a multistage, stratified probability sample design [7], and has been widely used to inform policy [18]. To date, there have been three rounds of Natsal, in 1990-'91 (Natsal-1), 1999-2001 (Natsal-2) and 2010-'12 (Natsal-3). Full details of the survey methods are reported elsewhere [6, 19, 20].

For this analysis, a LCA model was built on a combined dataset of participants from the three Natsal surveys (as opposed to building three separate models, one for each survey). Therefore, the population eligible for analysis was restricted to the eligibility criteria of Natsal-2, the survey round with the narrowest age-range (16–44 year-olds), and by the breadth of the earlier surveys (Natsal-1 focused only on HIV risk, Natsal-2 on STI risk more broadly, and Natsal-3 addressed broader themes of SRH). The sample was further restricted to only include individuals who were sexually active in the past year (determined by reporting any sexual partners during this time) to represent the subpopulation with current needs for SRH interventions and services. Men and women were modelled separately due to aforementioned differences in sexual lifestyles. Data preparation and analysis were conducted in R (4.2.1) [21].

### Model

**Fig. 1:**
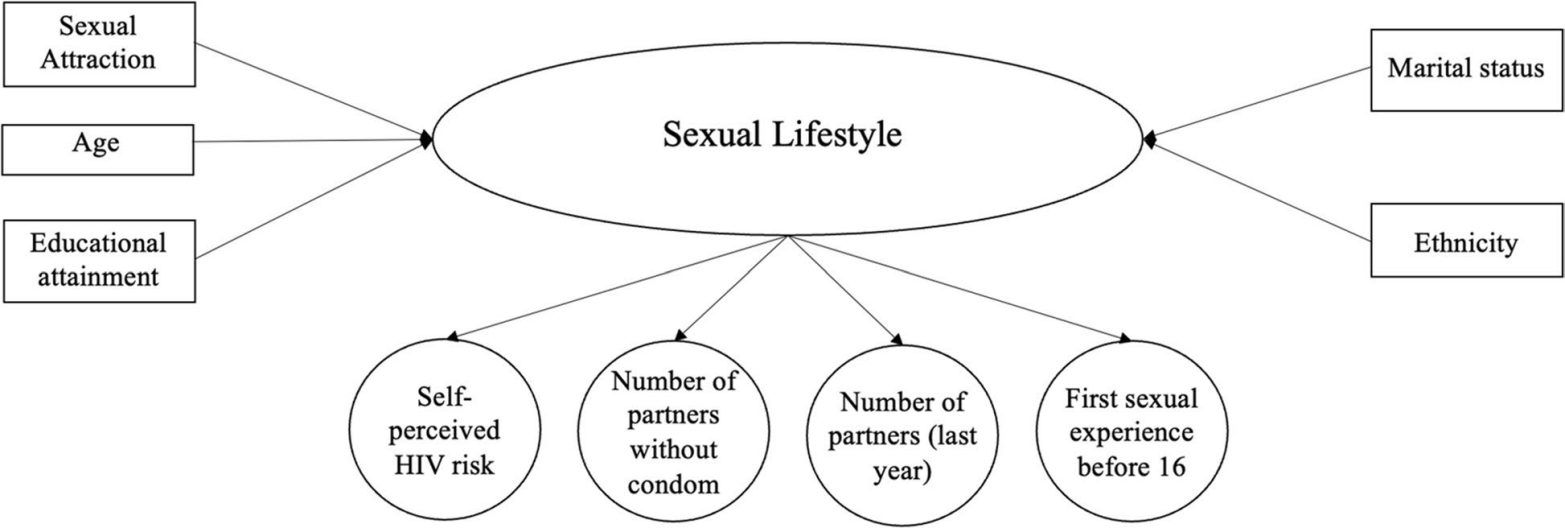
Diagrammatic representation of the Latent Class Analysis model used for our analysis. This model includes the latent variable being measured (sexual lifestyles) the manifest variables the model was built from and the covariate variables that were investigated

The selection of manifest variables was based on variables used by previous LCAs on Natsal data [4, 5] and examination of the literature for sexual lifestyles most likely to have implications for public health [2, 6, 12, 22]. Four manifest variables were selected: number of sexual partners (past year), number of partners without a condom (past year), age at first sex, and present self-perceived HIV risk. Self-perceived HIV risk has not been included in previous LCA models [5], but may have implications for behaviours resulting in the need for SRH intervention [22, 23]. Figure 1 represents the LCA model used for the analysis. The derivations of both manifest and covariate variables are reported in Additional File 1.

We used the Akaike Information Criterion (AIC) and the Bayesian Information Criterion (BIC) for model selection [24, 25], which perform well in identifying optimal class number [26–28].

To our knowledge, there is no established method for assessing change over time using LCA models. For our analysis, individuals were assigned a class based on the class for which their probability of membership was highest. Change over time was analysed using a multinomial regression analysis of class membership on year surveyed, generating risk ratios (RRs). Statistical significance for RRs was determined by whether 95% confidence intervals overlapped 1. The cohort was then split into original surveys, and a χ^2^ test was performed assessing the difference between the number of individuals assigned to each class for Natsal-2 and -3, compared to the expected number had class proportions not changed from Natsal-1. To understand for whom sexual lifestyles changed over time, a multinomial regression of class membership on sociodemographic characteristics was performed for each Natsal survey. Choice of covariates was informed by previous empirical research and models, and included age [4], marital status [29], sexual attraction (as the broadest measure of sexual orientation in contrast to sexual identity or experience, [30] and as the variable common across all three Natsal surveys), educational attainment [7] and ethnicity [22].

## Results

### Sample characteristics

Our study sample included 12,590 men and 17,235 women aged 16–44 who reported any sexual partners in the past year (i.e., were sexually active), with 11,738 individuals from Natsal-1, 9,690 from Natsal-2 and 8,397 from Natsal-3. Missing data was low (0.44% of values were missing), and no individuals had missing data for all manifest variables. Due to the risks of using data from different time points for imputation, and the low level of missingness, class allocation was made using membership probability from available data [5]. Sample characteristics are summarised in Table 1.

**Table 1:** **Sample characteristics,** by sex and Natsal survey

**Sociodemographic characteristic**	**Natsal-1 (%**^a^**)**		**Natsal-2 (%)**		**Natsal-3 (%)**		**Combined (Natsal-1, -2 and -3) (%)**	
	Men (*n* = 5081, 43.3%)	Women (*n* = 6657, 56.7%)	Men (*n* = 4104, 42.4%)	Women (*n* = 5586, 57.6%)	Men (*n* = 3405, 40.6%)	Women (*n* = 4992, 59.4%)	**Men (*n* = 12,590, 42.2%)**	**Women (*n* = 17,235, 57.8%)**
Age Category								
16–24	21.8	22.4	23.0	20.4	38.1^b^	45.1	**26.6**	**25.0**
25–34	41.5	43.8	39.5	41.7	40.6	33.7	**40.6**	**43.5**
35–44	36.7	33.8	37.5	37.9	21.3	21.3	**32.8**	**14.1**
Ethnicity								
White	95.0	95.2	88.6	90.0	87.5	87.7	**91.3**	**91.3**
Other Ethnic Groups	4.7	4.6	10.0^c^	9.8	12.3	12.1	**8.5**	**8.4**
Sexual attraction								
Exclusively opposite sex	94.2	94.1	90.0	87.1	92.2	82.6	**92.3**	**88.5**
Not exclusively opposite sex	5.7	5.8	9.9	12.7	7.6	17.4	**7.6**	**11.4**
Relationship Status								
Married/cohabiting	65.4	69.3	52.8	61.2	44.2	48.9	**55.5**	**60.8**
Not cohabiting/single	34.6	30.7	47.1	38.7	55.6	50.9	**44.4**	**39.1**
Highest educational qualification								
Degree-level	13.4	9.3	24.1	20.0	25.3	27.1	**20.1**	**18.0**
Below degree-level	69.1	67.7	59.5	63.6	64.6	63.5	**64.8**	**65.1**
No qualification	17.3	22.9	16.2	16.1	9.9	9.2	**14.9**	**16.7**
Number of partners in last year								
1	80.5	90.0	69.3	82.7	68.9	77.5	**73.7**	**84.0**
2 +	19.5	10.0	30.3	17.1	31.2	22.5	**26.2**	**15.9**
Number of partners without a condom in last year								
0	42.4	28.8	52.9	39.1	55.8	40.3	**49.4**	**35.5**
1	46.8	62.0	36.1	51.2	33.0	46.9	**39.6**	**54.1**
2 +	7.2	5.5	7.3	6.4	7.4	8.6	**7.3**	**6.7**
First sexual experience before 16								
16+	72.2	86.9	65.7	75.0	63.6	65.5	**67.8**	**76.8**
Before 16	27.8	13.1	34.3	25.0	36.4	34.5	**32.2**	**23.2**
Self-perceived HIV risk								
Not at-risk	68.1	74.2	56.6	67.4	63.6	73.4	**63.2**	**71.8**
At-risk	31.5	25.4	42.9	32.1	35.8	26.0	**36.4**	**27.8**

^a^Calculated from the number of individuals reporting the outcome divided by the number of men/women sampled from each survey. Due to small numbers of missing data, not all values add to 100%

^b^The higher proportions of young people in Natsal-3 are due to Natsal-3 oversampling people aged 16-34 years

^c^The higher proportions of ethnic minority groups in Natsal-2 are due to Natsal-2 oversampling people from key ethnic minority groups

### Model

For men and women, a three-class model was determined to be the best fit (Table 2), as the model with thelowest AIC and BIC values (Additional File 2)*.* Most men and women (41.3% and 60.9% respectively) were in Class 1, characterised by reporting one partner in the last year, a high probability of reporting condomless sex, and a high probability of being married/cohabiting (Additional File 3). The second largest class (32.5% of men, 23.4% of women; Class 2) also reported just one partner in the last year, but had a lower probability of reporting condomless sex and higher self-perceived HIV risk than those in Class 1. Finally, those in Class 3, corresponding to 26.2% of men and 15.7% of women, all reported 2 or more partners in the last year, with a relatively high probability of reporting condomless sex, relatively high self-perceived HIV risk, and a higher probability of first sex before 16 years of age. Self-reported number of partners in the last year was identified as the key characteristic differentiating Class 3 from the other two classes, indicated by conditional response probabilities of 1.00 for all classes with respect to number of sexual partners*.*

**Table 2:** Conditional Response Probabilities for each Latent Class. Conditional Response Probabilities of sexual lifestyles for men and women, with latent class prevalence for each class reported as a percentage next to the class. Bolded values represent those probabilities that indicate important characteristics of each class

**Men**	**Class 1 (41.3%)**	**Class 2 (32.5%)**	**Class 3 (26.2%)**
Number of partners in last year			
1	**1.00**	**1.00**	0.00
2 +	0.00	0.00	**1.00**
Number of partners without a condom in last year			
0	0.12	**0.90**	0.71
1	**0.88**	0.10	0.00
2 +	0.00	0.00	**0.29**
First sexual experience before 16			
16+	0.70	0.75	0.56
Before 16	0.30	0.25	**0.44**
Self-perceived HIV risk			
Not at-risk	**0.80**	0.66	0.32
At-risk	0.20	**0.34**	**0.68**
**Women**	**Class 1 (60.9%)**	**Class 2 (23.4%)**	**Class 3 (15.7%)**
Number of partners in last year			
1	**1.00**	**1.00**	0.00
2 +	0.00	0.00	**1.00**
Number of partners without a condom in last year			
0	0.31	**0.40**	0.55
1	**0.69**	0.60	0.01
2 +	0.00	0.00	**0.44**
First sexual experience before 16			
16+	0.81	0.78	0.60
Before 16	0.19	0.22	**0.40**
Self-perceived HIV risk			
Not at-risk	**0.97**	0.28	0.41
At-risk	0.02	**0.72**	**0.59**

### Have sexual lifestyles changed over time?

**Fig. 2:**
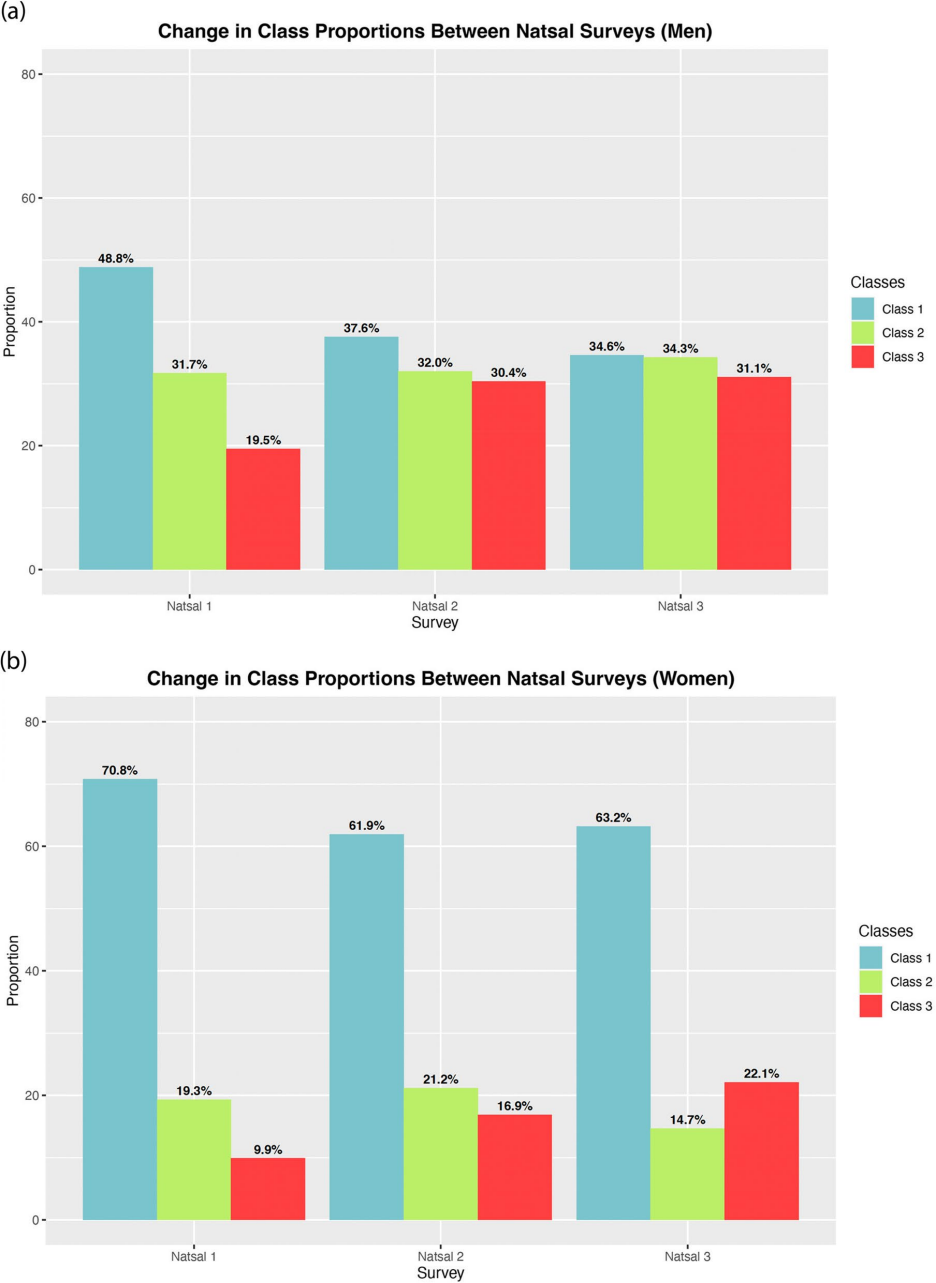
Change over time in Latent Class Proportions. The proportion of (**a**) men and (**b**) women assigned to each class across Natsal-1, Natsal-2 and Natsal-3

For both men (Fig. 2 (a)) and women (Fig. 2 (b)), there was an increase over time in the proportion of individuals in Class 3. Absolute change in proportion was similar for men and women between Natsal-1 and -3 (12.2 and 11.6 respectively), although the proportion of women in Class 3 was less than men at all time points.

A χ^2^ test investigating class proportions indicated a significant difference between the observed proportions in each latent class in Natsal-2 and -3 compared to the expected proportions if lifestyles had not changed since Natsal-1 for both men (χ^2^ = 739.49, *p *< 0.01) and women (χ^2^ = 1270.43, *p* < 0.01; see Additional File 4 for the probability table constructed for this analysis). In the multinomial regression model of class membership on survey sampled (Natsal-1, -2 or 3), being sampled in Natsal-2 or Natsal-3 was statistically significantly associated with a higher probability of being in Class 3 compared to Natsal-1 (Table 3).

**Table 3:** Multinomial Regression Analysis of class membership on survey number. Presented as relative risk ratios (RRRs). Bolded values indicate significance, identified by RRRs excluding 1.00

**Men**	Class 2	Class 3	**Women**	Class 2	Class 3
	RRRs	RRRs		RRRs	RRRs
Natsal-1	1.00	1.00	Natsal-1	1.00	1.00
Natsal-2	**1.30 (1.19–1.44)**	**2.03 (1.82–2.25)**	Natsal-2	**1.26 (1.15–1.38)**	**1.95 (1.75–2.17)**
Natsal-3	**1.53 (1.38–1.69)**	**2.25 (2.01–2.51)**	Natsal-3	**0.85 (0.77–0.94)**	**2.49 (2.24–2.77)**

### Among whom have sexual lifestyles changed?

 A multinomial regression analysis of class membership on sociodemographic characteristics found not cohabiting/being single corresponded with an increased probability of being in Class 3 for men and women (RRs greater than *10.00* for men and women across all time points), as did reporting any same-sex attraction (Additional File 5). Association with age was inconsistent across time points, although there was always a trend towards individuals in the youngest age category being more likely to be in Class 3. Although the association with ethnicity and being in Class 3 did not change over time, it did vary by sex, with men of ethnic groups other than white consistently being more likely to belong to Class 3 than white men. Ethnicity was not significantly associated with class membership for women.

## Discussion

### Principal findings

We have demonstrated the feasibility of LCA to: capture change over time in sexual lifestyles; and quantify *if* and *for* whom these lifestyles have changed in Britain between 1990 and 2010. To the authors’ knowledge, using LCA to assess change over time has not previously been performed in the context of SRH. We identified three classes of sexual lifestyles within the sexually active British population, predominantly driven by the number of sexual partners reported in the last year. We determined a statistically significant increase in the size of Class 3 from 1990–2010, consistent with findings from previous survey regression analyses that suggest sexual lifestyles considered to be associated with an increased probability of HIV transmission were at a low in 1990, following the emergence of HIV/AIDS [31].

In addition to demonstrating the potential of LCA to model change over time (our paper’s first objective), our findings also emphasise important facets of sexual lifestyles and how they have changed between 1990 and 2010 (our paper’s second objective). For example, our findings support the notion of significant differences in sexual lifestyles as reported by men and women [7]. Across all time points, there was a smaller proportion of women than men in Class 3. Previous LCA analyses have noted men being more likely than women to belong to latent classes  associated with STI/HIV transmission [4, 5], and survey analyses of the Natsal data have found men report a greater number of sexual partners and lifestyles associated with an increased probability of STI/HIV transmission than women (e.g., paying for sex; having new partners from outside of the UK) [2, 7]. In contrast, women have been found to report greater risk aversion than men [32]. However, in our study, the proportion of women assigned to Class 3 increased between 1990-2010, while the proportion of men in this class has possibly reached a plateau, consistent with little change in the proportion of men in Class 3 between 2000 and 2010, and empirical observations in the wider SRH literature reporting the number of sexual partners for men in the population possibly stabilising [7], indicating a potential shift in previously reported differences in sexual behaviour between sexes.

The strongest sociodemographic association with Class 3 membership was being single/not cohabiting, supporting the significance of both the number and type of sexual partners reported in driving class membership. Marital status has been identified as a significant correlate of sexual behaviour in previous LCA models [4, 5] and empirical work [7, 33], and evidence suggests that married people as a group have a lower probability of STI transmission than their single counterparts [25]. The association between age and class membership in our study was less pronounced than that observed by other studies, which report strong associations between young age and sexual lifestyles associated with HIV/STI transmission [7, 33]. These findings could in part reflect the correlation between age and marital status, rather than age being an independent correlate of sexual behaviour [33], supported by our sensitivity analyses indicating a consistently statistically significant association between younger age with class membership before adjusting for marital status (Additional File 6). The role of other demographic variables in understanding independent associations with sexual lifestyle is particularly acute for ethnicity, with some studies finding no independent correlation with the reporting of sexual behaviour [5, 22], despite a higher prevalence of STIs among particular ethnic groups [1], although this finding is likely to reflect healthcare-seeking behaviour,access-related factors and network factors that exist independent of individual-level *sexual* behaviour [34]. The relationship between sexual lifestyles and ethnicity for women was not significant in our analysis, but men of ethnic groups other than white were more likely to belong to Class 3 at all survey times, suggesting that differences in sexual lifestyles could account for some of the observed differences in STI prevalence at a population level. However, these findings are limited by the reduction of ethnicity to a binary variable in our analysis due to relatively small samples of ethnic minority groups in Natsal, as is the case with many surveys of the general population.

### Strengths and weaknesses

This study is the first, to our knowledge, to use LCA to explore change over time in the clustering of sexual lifestyles. Our sex-specific analyses also build upon established differences in behaviour between men and women [4]. There are methodological limitations associated with LCA, such as there being no single method for determining class number and a reliance on selecting manifest variables a priori. Our multinomial regression analysis based class allocation on highest probability of membership for an individual, and thus does not sufficiently capture those individuals with ambiguous class membership (i.e., 33% probability of membership for all classes). As software constraints of *poLCA* did not allow the use of complex survey analysis, our analysis was limited by using unweighted survey data, although reassuringly, our findings are consistent with previous studies that used complex survey analyses [5, 7].

### Strengths and weaknesses in relation to other studies

The relative strength of this study is the extension of previous LCA models conducted using Natsal data to investigate change over time, the methodology for which (particularly the use of a combined dataset for model fitting) is validated by our findings replicating previous LCA models and empirical findings [4, 5]. The use of a combined dataset for model generation ensured the classes derived were the same across all surveys to facilitate change-over-time analysis, however this method risked over-fitting the data to the survey with the most responses (Natsal-1). The analogous classes derived by our model and models constructed on Natsal-2 data alone [5] suggest the extent of this overfitting was minimal.

The primary limitation of our analysis compared to other studies was the availability of data common to all surveys, which excluded important aspects of sexual lifestyles (e.g., having new partners [5], and measures of different dimensions of sexual orientation aside from sexual attraction). Focusing the denominator for our analyses on the sexually active population means our findings are also likely to over-estimate sexual behaviours associated with STI/HIV transmission within the population as a whole. However, with around 80% of the British general population aged 16–74 years reporting partnered sexual activity in the past year in Natsal-3 [7], this focus does correspond to the vast majority and possibly those with greatest need for interventions. Data availability also meant our manifest variables were primarily based around sexual partner numbers and type, including variables that are strongly correlated (e.g., the number of sexual partners in the past year and the number of sexual partners reported without a condom in the past year [35], and between partner type and condom use, (although research shows that this latter relationship to be less clear [36])), which risks our model reflecting changes in these elements of sexual lifestyle rather than sexual lifestyles more broadly. However, previous LCA analyses have also found that partnership number is the primary driver of class membership [5]. A sensitivity analysis including same-sex experience as a manifest variable (reported in Additional File 7) found minimal change in class proportions from our model but generated classes that were deemed less insightful. An over-reliance on variables related to the number of sexual partners could also increase the risk of reporting bias influencing our findings [37]. It is also likely that manifest variables such as self-perceived HIV risk and their associated impact on sexual behaviour changed between 1990–2010. Our selected manifest variables also combine variables that correspond to behaviour both proximal and distal to the time of the survey, which may influence our findings, reflecting the challenge of capturing multidimensional concepts with complex interactions as is the case with sexual lifestyles. Finally, as the data used are from Natsal, a cross-sectional survey, rather than a cohort study, we are unable to draw conclusions regarding how individuals’ behaviour changes over time and over the lifecourse.

### Implications

This methodology gives a framework through which change over time can be investigated using LCA, which can answer novel questions in SRH and extend pre-existing models. Specifically, this methodology can be used in further rounds of Natsal (e.g., Natsal-COVID [38], Natsal-4 [39]), incorporating the greater diversity of data collected and allowing us to capture a more comprehensive picture of sexual lifestyles in the population. Our findings also support targeting public health interventions to those reporting higher numbers of sexual partners. This could include encouraging individuals to use their own number of sexual partners as a proxy for their own need to engage with SRH services, considered alongside their individual- and partnership-level prevention efforts to reduce occurrence of adverse outcomes (e.g., whether condoms are used, and if so, whether they are used correctly). Such consideration could help inform decisions about screening engagement [5].

### Unanswered questions

Survey eligibility criteria and data availability restricted us from making conclusions about behaviour in specific populations. Our findings provide no insight into the SRH of gender-diverse individuals, which are a community who are often overlooked in SRH research and policy [40], due to previous rounds of Natsal not collecting data on gender identity. As subsequent surveys, including but not limited to Natsal, incorporate more comprehensive measures of gender-identity, future analyses will be able to reflect these. Similarly, restriction to 16–44-year-olds excluded older individuals who are an important butunder-represented group within SRH research [41]. Our analysis also does not represent a complete picture of sexual lifestyle, with factors such as sexual networks and partner behaviour having important impacts on individual behaviour and likelihood of experiencing adverse SRH outcomes [33, 42]. Our analysis also only represents change over time until 2010, so it is important to extend these findings to identify more recent changes in sexual lifestyles and SRH service need to inform future service delivery and public health messaging[43, 44], especially given the impacts of the COVID-19 pandemic on the population's SRH [45]. As this work has shown, LCA is a useful tool for doing so by providing a more sophisticated approach to understanding sexual lifestyles and other complex behavioural phenomena.

### Supplementary Information

**Additional file 1.  **Derivation of the manifest and covariate variables used in our analysis. Reports variable name, the specific details of these variables and the categories within each variable to which individuals were assigned.

**Additional file 2. **Information Criterion for Latent Class Analysis (LCA) models. The Akaike Information Criterion (AIC) and Bayesian Information Criterion (BIC) of LCA models with different class sizes (between 2 and 4 classes) for men and women.

**Additional file 3. **Marital Status of Latent Classes. Reported for the combined cohort of Natsal 1, 2 and 3.

**Additional file 4. **χ2 test probability table. Reporting the numbers of individuals assigned to each latent class for Natsal 2 and Natsal 3, the numbers that would be expected to be assigned to each latent class if class proportions from Natal 1 had not change, and the results of a χ2 test investigating the significance of this difference.

**Additional file 5. **Multinomial Regression Analysis of Class Membership on Sociodemographic Characteristics Including age, sexual attraction, ethnicity, marital status, and educational attainment (mutually adjusted for one another) for each survey, separated by sex. Reported as adjusted relative risk ratios (RRRs), alongside the proportion of individuals within each class who reported each characteristic. Due to small amounts of missing data, not all proportions add to 100%. Bolded values signify statistical significance, determined by RRRs excluding 1.00.

**Additional file 6. **Adjusted and unadjusted risk ratios for class membership and age for Natsal surveys. RRRs for assignment to Classes 2 and 3 for Natsal 2 and 3 compared to Natsal 1. Adjusted RRRs are adjusted for marital status, ethnicity, same-sex attraction, and educational attainment.

**Additional file 7. **Sensitivity Analysis including same sex experience as a manifest variable. Model fit statistics (AIC and BIC Values) indicating a two-class model is the most viable, the characteristics of the classes as specified by conditional response probabilities and the proportions of men and women assigned to each class in Natsal 1, 2 and 3.

## Data Availability

The datasets supporting the conclusions of this article are available in the UK Data Service repository, SN3434 (http://doi.org/10.5255/UKDA-SN-3434–1), SN5223 (http://doi.org/10.5255/UKDA-SN-5223–1) and SN7799 (http://doi.org/10.5255/UKDA-SN-7799–2).
