# Supplementary material for: Examining changes in sexual lifestyles in Britain between 1990–2010: a latent class analysis approach
Source: BMC Public Health. 2024 Feb 3;24:366. doi: 10.1186/s12889-024-17850-1 (PMC10837868; doi:10.1186/s12889-024-17850-1)
Supplement: Supplementary file 1 — Additional file 1. Derivation of the manifest and covariate variables used in our analysis. Reports variable name, the specific details of these variables and the categories within each variable to which individuals were assigned. [file 12889_2024_17850_MOESM1_ESM.docx]

***Additional File 1 -* Derivation of the manifest and covariate variables used in our analysis**. Reports variable name, the specific details of these variables and the categories within each variable to which individuals were assigned.

| **Variable** | **Details** | **Grouping** |
| --- | --- | --- |
|  |  |  |
| **LCA Manifest Variables** | | |
| Number of sexual partners in past year | A binary variable derived from the sum of number of reported opposite-sex and same-sex partners | 1; 2+ |
| Number of partners in the last year without a condom | *2+;* if reported not using a condom in the past year, reported opposite/same sex vaginal/anal sex in the last year and reported 2+ partners in the last year  *1;* if reported not using a condom in the past year, reported opposite/same sex vaginal/anal sex in the last year and reported 1 partner in the last year  *0*; if reported using a condom in the past year | 2+; 1; 0 |
|  |  |  |
| First sexual intercourse before 16 | *Yes;* if first reported opposite and/or same-sex intercourse before the age 16 | Yes; No |
| Self-perceived HIV risk | *At-risk;* if perceived themselves to be greatly/quite/not really at risk of HIV acquisition  *Not at risk;* if perceived themselves to be not at all at risk of HIV | At-risk; Not at-risk |
| **Multinomial Regression Covariates** | | |
| Sexual attraction | *Only same-sex attraction;* if reported exclusive attraction to the opposite sex  *Not only same-sex attraction*; if reported anything but exclusive attraction to the opposite sex | Only same-sex attraction; Not only same-sex attraction |
| Ethnicity | Self-reported ethnicity, separated into binary categories of white and other ethnic groups | White; Other Ethnic Groups |
| Highest qualification attained. | Self-reported highest educational qualification attained | Degree-level; below degree-level; no qualifications |
|  |  |  |
| Marital status | Self-reported marital status | Married/cohabiting; single/not cohabiting |
| Age category | Age of participants | 16-24; 25-34; 35-44 |
