# Supplementary material for: Examining changes in sexual lifestyles in Britain between 1990–2010: a latent class analysis approach
Source: BMC Public Health. 2024 Feb 3;24:366. doi: 10.1186/s12889-024-17850-1 (PMC10837868; doi:10.1186/s12889-024-17850-1)

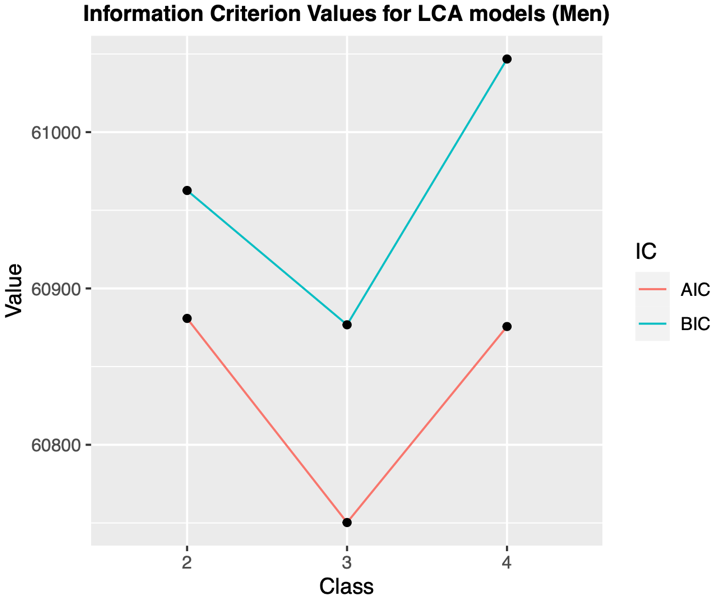
**Additional File 2 - Information Criterion for Latent Class Analysis (LCA) models**. The Akaike Information Criterion (AIC) and Bayesian Information Criterion (BIC) of LCA models with different class sizes (between 2 and 4 classes) for men and women.


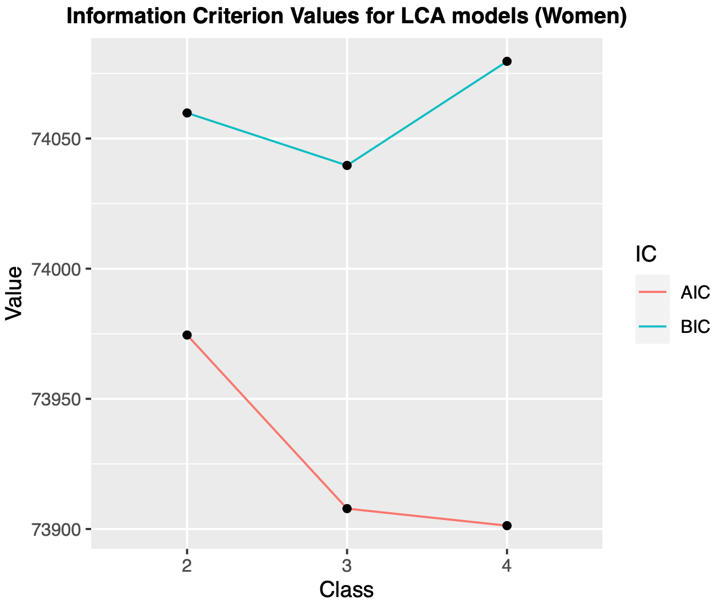

Supplement: Supplementary file 2 — Additional file 2. Information Criterion for Latent Class Analysis (LCA) models. The Akaike Information Criterion (AIC) and Bayesian Information Criterion (BIC) of LCA models with different class sizes (between 2 and 4 classes) for men and women. [file 12889_2024_17850_MOESM2_ESM.docx]
