# Supplementary material for: Examining changes in sexual lifestyles in Britain between 1990–2010: a latent class analysis approach
Source: BMC Public Health. 2024 Feb 3;24:366. doi: 10.1186/s12889-024-17850-1 (PMC10837868; doi:10.1186/s12889-024-17850-1)
Supplement: Supplementary file 3 — Additional file 3. Marital Status of Latent Classes. Reported for the combined cohort of Natsal 1, 2 and 3. [file 12889_2024_17850_MOESM3_ESM.docx]

***Additional File 3 – Marital Status of Latent Classes****.* Reported for the combined cohort of Natsal 1, 2 and 3.

| **Men** | **Class 1 (%)** | **Class 2 (%)** | **Class 3 (%)** |
| --- | --- | --- | --- |
| Married/cohabiting | 79.3 | 56.7 | 16.9 |
| Single/not cohabiting | 20.7 | 43.3 | 83.1 |
| **Women** |  |  |  |
| Married/cohabiting | 74.1 | 51.4 | 16.6 |
| Single/not cohabiting | 25.9 | 48.6 | 83.4 |
