# Supplementary material for: Examining changes in sexual lifestyles in Britain between 1990–2010: a latent class analysis approach
Source: BMC Public Health. 2024 Feb 3;24:366. doi: 10.1186/s12889-024-17850-1 (PMC10837868; doi:10.1186/s12889-024-17850-1)
Supplement: Supplementary file 4 — Additional file 4. χ2 test probability table. Reporting the numbers of individuals assigned to each latent class for Natsal 2 and Natsal 3, the numbers that would be expected to be assigned to each latent class if class proportions from Natal 1 had not change, and the results of a χ2 test investigating the significance of this difference. [file 12889_2024_17850_MOESM4_ESM.docx]

***Additional File 4 - χ^2^ test probability table.*** *Reporting the numbers of individuals assigned to each latent class for Natsal 2 and Natsal 3, the numbers that would be expected to be assigned to each latent class if class proportions from Natal 1 had not change, and the results of a χ^2^ test investigating the significance of this difference.*

| **Men** | Class 1 | Class 2 | Class 3 |
| --- | --- | --- | --- |
| Natsal 1 observed values | 2480 | 1610 | 991 |
| Natsal 2 observed values | 1543 | 1313 | 1248 |
| Natsal 2 expected values^^[[1]](#footnote-1)^^ | 2002 | 1301 | 800 |
| **Observed - expected** | **-459** | **12** | **448** |
| Natsal 3 observed values | 1178 | 1168 | 1059 |
| Natsal 3 expected values^^[[2]](#footnote-2)^^ | 1662 | 1079 | 664 |
| **Observed - expected** | **-484** | **89** | **395** |
| χ^2^ value (d.f. = 2) | **739.49 (*p < 0.01)*** |  |  |

| **Women** | Class 1 | Class 2 | Class 3 |
| --- | --- | --- | --- |
| Natsal 1 observed values | 4713 | 1285 | 659 |
| Natsal 2 observed values | 3458 | 1184 | 944 |
| Natsal 2 expected values | 3955 | 1078 | 553 |
| **Observed - expected** | **-497** | **106** | **441** |
| Natsal 3 observed values | 3155 | 734 | 1103 |
| Natsal 3 expected values | 3534 | 963 | 494 |
| **Observed - expected** | **-379** | **-229** | **609** |
| χ^2^ value (d.f. = 2) | **1270.43 (*p < 0.01)*** |  |  |

1. Expected values are calculated as following: *proportion of Natsal 1 population belonging to the class (found in Figure 2) x number surveyed in Natsal 2 (9,690)* [↑](#footnote-ref-1)
2. Expected values are calculated as following: *proportion of Natsal 1 population belonging to the class (found in Figure 2) x number surveyed in Natsal 3 (8,397)* [↑](#footnote-ref-2)
