# Supplementary material for: Examining changes in sexual lifestyles in Britain between 1990–2010: a latent class analysis approach
Source: BMC Public Health. 2024 Feb 3;24:366. doi: 10.1186/s12889-024-17850-1 (PMC10837868; doi:10.1186/s12889-024-17850-1)
Supplement: Supplementary file 5 — Additional file 5. Multinomial Regression Analysis of Class Membership on Sociodemographic Characteristics Including age, sexual attraction, ethnicity, marital status, and educational attainment (mutually adjusted for one another) for each survey, separated by sex. Reported as adjusted relative risk ratios (RRRs), alongside the proportion of individuals within each class who reported each characteristic. Due to small amounts of missing data, not all proportions add to 100%. Bolded values signify statistical significance, determined by RRRs excluding 1.00. [file 12889_2024_17850_MOESM5_ESM.docx]

***Additional File 5 - Multinomial Regression Analysis of Class Membership on Sociodemographic Characteristics*** Including age, sexual attraction, ethnicity, marital status, and educational attainment (mutually adjusted for one another) for each survey, separated by sex. Reported as adjusted relative risk ratios (RRRs), alongside the proportion of individuals within each class who reported each characteristic. Due to small amounts of missing data, not all proportions add to 100%. Bolded values signify statistical significance, determined by RRRs excluding 1.00.

| **Natsal-1 (Men)** | Class 1 | Class 2 | | Class 3 | | |
| --- | --- | --- | --- | --- | --- | --- |
|  | % | % | Adjusted RRR | | % | Adjusted RRR |
| Age |  |  |  | |  |  |
| 16-24 | 13.4 | 24.4 | **1.27 (1.05-1.54)** | | 71.1 | 1.18 (0.94-1.47) |
| 25-34 | 40.3 | 45.3 | 1.00 | | 12.6 | 1.00 |
| 35-44 | 46.3 | 30.2 | **0.63 (0.54-0.73)** | | **16.0** | **0.74 (0.60-0.92)** |
| Ethnicity |  |  |  | |  |  |
| White | 95.7 | 94.5 | 1.00 | | 94.1 | 1.00 |
| Other Ethnic Groups | 4.0 | 5.1 | 1.35 (0.99-1.83) | | 5.5 | **1.72 (1.16-2.53)** |
| Sexual attraction |  |  |  | |  |  |
| Exclusively opposite sex | 96.0 | 94.5 | 1.00 | | 89.2 | 1.00 |
| Not exclusively opposite sex | 4.0 | 5.5 | 1.21 (0.89-1.65) | | 10.6 | **2.13 (1.53-2.97)** |
| Relationship Status |  |  |  | |  |  |
| Married/cohabiting | 82.6 | 65.1 | 1.00 | | 22.7 | 1.00 |
| Not cohabiting/single | 17.4 | 34.9 | **2.08 (1.76-2.44)** | | 77.2 | **13.86 (11.38-16.90)** |
| Highest educational qualification |  |  |  | |  |  |
| Degree-level | 11.5 | 16.9 | 1.00 | | 12.6 | 1.00 |
| Below degree-level | 67.3 | 70.7 | **0.65 (0.53-0.78)** | | 71.1 | 0.88 (67-1.15) |
| No qualification | 21.1 | 12.3 | **0.38 (0.30-0.49)** | | 16.0 | **0.66 (0.48-0.91)** |
| **Natsal-1 (Women)** |  |  |  | |  |  |
| Age |  |  |  | |  |  |
| 16-24 | 18.2 | 26.6 | 1.15 (0.97-1.35) | | 43.9 | **1.58 (1.28-1.94)** |
| 25-34 | 44.7 | 43.9 | 1.00 | | 37.2 | 1.00 |
| 35-44 | 37.1 | 29.5 | 0.91 (0.78-1.05) | | 18.9 | 0.81 (0.64-1.03) |
| Ethnicity |  |  |  | |  |  |
| White | 95.6 | 93.8 | 1.00 | | 94.4 | 1.00 |
| Other Ethnic Groups | 4.1 | 5.8 | 1.29 (0.97-1.71) | | 5.1 | 1.06 (0.71-1.59) |
| Sexual attraction |  |  |  | |  |  |
| Exclusively opposite sex | 95.5 | 92.5 | 1.00 | | 87.3 | 1.00 |
| Not exclusively opposite sex | 4.4 | 7.5 | **1.59 (1.22-2.06)** | | 12.6 | **2.92 (2.15-3.97)** |
| Relationship Status |  |  |  | |  |  |
| Married/cohabiting | 79.4 | 56.4 | 1.00 | | 22.5 | 1.00 |
| Not cohabiting/single | 20.6 | 43.6 | **2.85 (2.48-3.27)** | | 77.5 | **11.30 (9.20-13.88)** |
| Highest educational qualification |  |  |  | |  |  |
| Degree-level | 8.1 | 13.1 | 1.00 | | 10.6 | 1.00 |
| Below degree-level | 67.3 | 67.4 | **0.61 (0.50-0.75)** | | 71.3 | 0.75 (0.55-1.02) |
| No qualification | 24.5 | 19.4 | **0.49 (0.39-0.62)** | | 18.2 | **0.56 (0.39-0.78)** |
| **Natsal-2 (Men)** | Class 1 | Class 2 | | Class 3 | | |
|  | % | % | Adjusted RRR | | % | Adjusted RRR |
| Age |  |  |  | |  |  |
| 16-24 | 6.7 | 27.6 | **3.17 (2.42-4.13)** | | 38.3 | **2.65 (2.02-3.47)** |
| 25-34 | 39.0 | 41.3 | 1.00 | | 38.4 | 1.00 |
| 35-44 | 54.3 | 31.2 | **0.60 (0.51-0.71)** | | 23.3 | **0.58 (0.47-0.71)** |
| Ethnicity |  |  |  | |  |  |
| White | 92.5 | 89.3 | 1.00 | | 86.9 | 1.00 |
| Other Ethnic Groups | 7.2 | 10.6 | **1.43 (1.09-1.88)** | | 13.0 | **1.81 (1.34-2.45)** |
| Sexual attraction |  |  |  | |  |  |
| Exclusively opposite sex | 92.5 | 92.1 | 1.00 | | 84.7 | 1.00 |
| Not exclusively opposite sex | 7.3 | 7.8 | 0.97 (0.72-1.30) | | 15.3 | **2.07 (1.54-2.76)** |
| Relationship Status |  |  |  | |  |  |
| Married/cohabiting | 79.0 | 55.0 | 1.00 | | 18.0 | 1.00 |
| Not cohabiting/single | 20.7 | 44.9 | **2.05 (1.71-2.47)** | | 81.9 | **11.73 (9.59-14.36)** |
| Highest educational qualification |  |  |  | |  |  |
| Degree-level | 20.0 | 30.0 | 1.00 | | 23.0 | 1.00 |
| Below degree-level | 59.5 | 57.1 | **0.51 (0.42-0.62)** | | 61.9 | **0.70 (0.56-0.88)** |
| No qualification | 20.1 | 12.7 | **0.42 (0.33-0.54)** | | 15.0 | **0.65 (0.49-0.86)** |
| **Natsal-2 (Women)** |  |  |  | |  |  |
| Age |  |  |  | |  |  |
| 16-24 | 14.6 | 23.7 | **1.27 (1.05-1.54)** | | 37.8 | **1.49 (1.22-1.82)** |
| 25-34 | 42.2 | 41.6 | 1.00 | | 39.8 | 1.00 |
| 35-44 | 43.1 | 34.7 | 0.94 (0.80-1.10) | | 22.4 | 0.72 (0.59-0.88) |
| Ethnicity |  |  |  | |  |  |
| White | 90.8 | 87.2 | 1.00 | | 90.5 | 1.00 |
| Other Ethnic Groups | 9.1 | 12.4 | **1.25 (1.01-1.56)** | | 9.2 | 0.83 (0.63-1.09) |
| Sexual attraction |  |  |  | |  |  |
| Exclusively opposite sex | 90.6 | 83.0 | 1.00 | | 79.4 | 1.00 |
| Not exclusively opposite sex | 9.2 | 16.9 | **1.85 (1.51-2.26)** | | 20.5 | **2.58 (2.07-3.23)** |
| Relationship Status |  |  |  | |  |  |
| Married/cohabiting | 76.0 | 50.5 | 1.00 | | 20.6 | 1.00 |
| Not cohabiting/single | 23.9 | 49.4 | **3.03 (2.61-3.51)** | | 79.2 | **10.70 (8.89-12.89)** |
| Highest educational qualification |  |  |  | |  |  |
| Degree-level | 18.0 | 26.3 | 1.00 | | 19.5 | 1.00 |
| Below degree-level | 64.6 | 59.9 | **0.59 (0.50-0.70)** | | 64.4 | **0.76 (0.61-0.94)** |
| No qualification | 17.1 | 13.5 | **0.49 (0.39-0.61)** | | 15.6 | **0.65 (0.50-0.86)** |
| **Natsal-3 (Men)** | Class 1 | Class 2 | | Class 3 | | |
|  | % | % | Adjusted RRR | | % | Adjusted RRR |
| Age |  |  |  | |  |  |
| 16-24 | 18.7 | 41.3 | **1.87 (1.48-2.35)** | | 56.1 | **1.59 (1.24-2.03)** |
| 25-34 | 46.4 | 42.1 | 1.00 | | 32.5 | 1.00 |
| 35-44 | 34.8 | 16.6 | **0.58 (0.47-0.72)** | | 11.3 | **0.67 (0.51-0.90)** |
| Ethnicity |  |  |  | |  |  |
| White | 88.5 | 88.1 | 1.00 | | 85.6 | 1.00 |
| Other Ethnic Groups | 11.1 | 11.9 | 1.15 (0.89-1.50) | | 14.3 | **1.73 (1.28-2.34)** |
| Sexual attraction |  |  |  | |  |  |
| Exclusively opposite sex | 95.1 | 93.3 | 1.00 | | 87.9 | 1.00 |
| Not exclusively opposite sex | 4.9 | 6.7 | 1.17 (0.82-1.69) | | 12.1 | **2.06 (1.43-2.99)** |
| Relationship Status |  |  |  | |  |  |
| Married/cohabiting | 72.2 | 46.9 | 1.00 | | 10.0 | 1.00 |
| Not cohabiting/single | 27.6 | 53.0 | **2.01 (1.64-2.45)** | | 89.9 | **12.21 (13.30-22.27)** |
| Highest educational qualification |  |  |  | |  |  |
| Degree-level | 26.8 | 28.7 | 1.00 | | 19.7 | 1.00 |
| Below degree-level | 61.0 | 64.3 | **0.75 (0.61-0.92)** | | 69.0 | 1.04 (0.81-1.33) |
| No qualification | 11.8 | 6.8 | **0.47 (0.34-0.66)** | | 11.2 | 1.60 (0.77-1.60) |
| **Natsal-3 (Women)** |  |  |  | |  |  |
| Age |  |  |  | |  |  |
| 16-24 | 26.1 | 33.2 | 1.03 (0.84-1.27) | | 55.7 | **1.44 (1.20-1.72)** |
| 25-34 | 48.9 | 46.4 | 1.00 | | 33.3 | 1.00 |
| 35-44 | 25.0 | 20.5 | 0.98 (0.79-1.21) | | 11.1 | 0.90 (0.70-1.15) |
| Ethnicity |  |  |  | |  |  |
| White | 88.6 | 84.0 | 1.00 | | 87.7 | 1.00 |
| Other Ethnic Groups | 11.2 | 16.0 | **1.57 (1.25-1.98)** | | 12.1 | 1.26 (0.99-1.61) |
| Sexual attraction |  |  |  | |  |  |
| Exclusively opposite sex | 86.1 | 81.0 | 1.00 | | 73.7 | 1.00 |
| Not exclusively opposite sex | 13.9 | 19.0 | **1.45 (1.16-1.80)** | | 26.3 | **2.33 (1.92-2.83)** |
| Relationship Status |  |  |  | |  |  |
| Married/cohabiting | 63.8 | 43.9 | 1.00 | | 9.6 | 1.00 |
| Not cohabiting/single | 36.1 | 55.9 | **2.31 (1.93-2.76)** | | 90.0 | **14.06 (11.23-17.61)** |
| Highest educational qualification |  |  |  | |  |  |
| Degree-level | 28.9 | 31.8 | 1.00 | | 19.0 | 1.00 |
| Below degree-level | 61.8 | 60.0 | **0.81 (0.67-0.97)** | | 70.5 | **1.22 (1.00-1.49)** |
| No qualification | 9.2 | 8.2 | **0.70 (0.51-0.96)** | | 9.9 | 1.09 (0.80-1.46) |
