# Supplementary material for: Examining changes in sexual lifestyles in Britain between 1990–2010: a latent class analysis approach
Source: BMC Public Health. 2024 Feb 3;24:366. doi: 10.1186/s12889-024-17850-1 (PMC10837868; doi:10.1186/s12889-024-17850-1)
Supplement: Supplementary file 6 — Additional file 6. Adjusted and unadjusted risk ratios for class membership and age for Natsal surveys. RRRs for assignment to Classes 2 and 3 for Natsal 2 and 3 compared to Natsal 1. Adjusted RRRs are adjusted for marital status, ethnicity, same-sex attraction, and educational attainment. [file 12889_2024_17850_MOESM6_ESM.docx]

***Additional File 6 – Adjusted and unadjusted risk ratios for class membership and age for Natsal surveys****. RRRs for assignment to Class 2 and Class 3 for Natsal 2 and 3 compared to Natsal 1. Adjusted RRRs are adjusted for marital status, ethnicity, same-sex attraction and educational attainment.*

| **Natsal 1 (Men)** | Class 2 | | Class 3 | | | | |  |
| --- | --- | --- | --- | --- | --- | --- | --- | --- |
|  | RRR | aRRR^^[[1]](#footnote-1)^^ | | RRR | | | aRRR | |
| Age |  |  | |  | | |  | |
| 16-24 | **1.62 (1.36-1.94)** | **1.27 (1.05-1.54)** | | **3.05 (2.53-3.68)** | | | 1.18 (0.94-1.47) | |
| 25-34 | 1.00 | 1.00 | | 1.00 | | | 1.00 | |
| 35-44 | **0.58 (0.50-0.67)** | **0.63 (0.54-0.73)** | | **0.53 (0.44-0.63)** | | | **0.74 (0.60-0.92)** | |
| **Natsal 1 (Women)** |  | |  | | | | |  |
| Age |  |  | |  | |  | | |
| 16-24 | **1.49 (1.27-1.74)** | 1.15 (0.97-1.35) | | **2.90 (2.40-3.49)** | | **1.58 (1.28-1.94)** | | |
| 25-34 | 1.00 | 1.00 | | 1.00 | | 1.00 | | |
| 35-44 | **0.81 (0.70-0.94)** | 0.91 (0.78-1.05) | | **0.61 (0.49-0.77)** | | 0.81 (0.64-1.03) | | |
| **Natsal 2 (Men)** |  | |  | | | | |  |
| Age |  |  | |  |  | | | |
| 16-24 | **3.90 (3.05-5.00)** | **3.17 (2.42-4.13)** | | **5.83 (4.56-7.45)** | | **2.65 (2.02-3.47)** | | |
| 25-34 | 1.00 | 1.00 | | 1.00 | | 1.00 | | |
| 35-44 | **0.54 (0.46-0.64)** | **0.60 (0.51-0.71)** | | **0.44 (0.37-0.52)** | | **0.58 (0.47-0.71)** | | |
| **Natsal 2 (Women)** |  | |  | | | | |  |
| Age |  |  | |  | | |  | |
| 16-24 | **1.65 (1.38-1.97)** | **1.27 (1.05-1.54)** | | **2.75 (2.30-3.28)** | | | **1.49 (1.22-1.82)** | |
| 25-34 | 1.00 | 1.00 | | 1.00 | | | 1.00 | |
| 35-44 | **0.81 (0.70-0.95)** | 0.94 (0.80-1.10) | | **0.55 (0.46-0.66)** | | | 0.72 (0.59-0.88) | |
| **Natsal 3 (Men)** |  | |  | | | | |  |
| Age |  |  | |  | | |  | |
| 16-24 | **2.42 (1.99-2.97)** | **1.87 (1.48-2.35)** | | **4.27 (3.48-5.25)** | | | **1.59 (1.24-2.03)** | |
| 25-34 | 1.00 | 1.00 | | 1.00 | | | 1.00 | |
| 35-44 | **0.53 (0.43-0.65)** | **0.58 (0.47-0.72)** | | **0.47 (0.36-0.59)** | | | **0.67 (0.51-0.90)** | |
| **Natsal 3 (Women)** |  | |  | | | | |  |
| Age |  |  | |  | | |  | |
| 16-24 | **1.34 (1.11-1.61)** | 1.03 (0.84-1.27) | | **3.13 (2.68-3.65)** | | | **1.44 (1.20-1.72)** | |
| 25-34 | 1.00 | 1.00 | | 1.00 | | | 1.00 | |
| 35-44 | 0.86 (0.70-1.06) | 0.98 (0.79-1.21) | | **0.65 (0.52-0.81)** | | | 0.90 (0.70-1.15) | |

1. Adjusted for marital status, ethnicity, same-sex attraction and educational attainment. [↑](#footnote-ref-1)
