# Supplementary material for: Examining changes in sexual lifestyles in Britain between 1990–2010: a latent class analysis approach
Source: BMC Public Health. 2024 Feb 3;24:366. doi: 10.1186/s12889-024-17850-1 (PMC10837868; doi:10.1186/s12889-024-17850-1)
Supplement: Supplementary file 7 — Additional file 7. Sensitivity Analysis including same sex experience as a manifest variable. Model fit statistics (AIC and BIC Values) indicating a two-class model is the most viable, the characteristics of the classes as specified by conditional response probabilities and the proportions of men and women assigned to each class in Natsal 1, 2 and 3. [file 12889_2024_17850_MOESM7_ESM.docx]

***Additional File 7 – Sensitivity Analysis including same sex experience as a manifest variable****. Model fit statistics (AIC and BIC Values) indicating a two-class model is the most viable, the characteristics of the classes as specified by conditional response probabilities and the proportions of men and women assigned to each class in Natsal 1, 2 and 3.*

| **Men** | AIC Value | BIC Value |
| --- | --- | --- |
| 1 class | 75868.27 | 75912.91 |
| **2 classes**^^[[1]](#footnote-1)^^ | **67936.86** | **68033.59** |
| 3 classes | 67401.04 | 67539.85 |
| 4 classes | 67277.33 | 67478.23 |
| 5 classes | 67277.67 | 67530.65 |
| **Women** | AIC Value | BIC Value |
| 1 class | 94728.74 | 94775.27 |
| **2 classes** | **85064.32** | **85165.13** |
| 3 classes | 83552.04 | 83707.14 |
| 4 classes | 83389.92 | 83599.30 |
| 5 classes | 83470.51 | 83734.17 |

| **Men** | Latent Class 1 (67.48%) | Latent Class 2 (32.52%) |
| --- | --- | --- |
| Number of partners in last year |  |  |
| 1 | 1.00 | 0.00 |
| 2+ | 0.00 | 1.00 |
| Number of partners without a condom in last year |  |  |
| 0 | 0.44 | 0.71 |
| 1 | 0.56 | 0.00 |
| 2+ | 0.00 | 0.29 |
| First sexual experience before 16 |  |  |
| After 16 | 0.72 | 0.56 |
| Before 16 | 0.28 | 0.44 |
| Self-perceived HIV risk |  |  |
| Not at-risk | 0.74 | 0.32 |
| At-risk | 0.26 | 0.68 |
| Ever had same sex experience |  |  |
| No | 0.94 | 0.86 |
| Yes | 0.06 | 0.14 |
| **Women** | Latent Class 1 (84.1%) | Latent Class 2 (15.9%) |
| Number of partners in last year |  |  |
| 1 | 1.00 | 0.00 |
| 2+ | 0.00 | 1.00 |
| Number of partners without a condom in last year |  |  |
| 0 | 0.33 | 0.55 |
| 1 | 0.67 | 0.01 |
| 2+ | 0.00 | 0.43 |
| First sexual experience before 16 |  |  |
| After 16 | 0.80 | 0.60 |
| Before 16 | 0.20 | 0.40 |
| Self-perceived HIV risk |  |  |
| Not at-risk | 0.78 | 0.42 |
| At-risk | 0.22 | 0.58 |
| Ever had same sex experience |  |  |
| No | 0.92 | 0.76 |
| Yes | 0.08 | 0.24 |

| **Men** | *Non-risky* | *Risky* |
| --- | --- | --- |
| Natsal 1 (5081) | 80.5 | 19.5 |
| Natsal 2 (4104) | 69.6 | 30.4 |
| Natsal 3 (3405) | 68.9 | 31.1 |

| **Women** | *Non-risky* | *Risky* |
| --- | --- | --- |
| Natsal 1 (6657) | 90.0 | 10.0 |
| Natsal 2 (5586) | 82.9 | 17.1 |
| Natsal 3 (4992) | 77.5 | 22.5 |

1. A two-class model was selected here due to the small percentage difference in information criterion values with the addition of other classes, and as class number increased in this model, the additional classes were small and based on very specific demographic profiles that were deemed less clinically informative than a those generated by a two-class model. [↑](#footnote-ref-1)
